# Sources of Heavy Metals in Surface Sediments and an Ecological Risk Assessment from Two Adjacent Plateau Reservoirs

**DOI:** 10.1371/journal.pone.0102101

**Published:** 2014-07-10

**Authors:** Binbin Wu, Guoqiang Wang, Jin Wu, Qing Fu, Changming Liu

**Affiliations:** 1 College of Water Sciences, Beijing Normal University, Key Laboratory of Water and Sediment Sciences, Ministry of Education, Beijing, China; 2 Chinese Research Academy of Environmental Sciences, Beijing, China; NIEHS/NIH, United States of America

## Abstract

The concentrations of heavy metals (mercury (Hg), cadmium (Cd), lead (Pb), chromium (Cr), copper (Cu) and arsenic (As)) in surface water and sediments were investigated in two adjacent drinking water reservoirs (Hongfeng and Baihua Reservoirs) on the Yunnan-Guizhou Plateau in Southwest China. Possible pollution sources were identified by spatial and statistical analyses. For both reservoirs, Cd was most likely from industrial activities, and As was from lithogenic sources. For the Hongfeng Reservoir, Pb, Cr and Cu might have originated from mixed sources (traffic pollution and residual effect of former industrial practices), and the sources of Hg included the inflows, which were different for the North (industrial activities) and South (lithogenic origin) Lakes, and atmospheric deposition resulting from coal combustion. For the Baihua Reservoir, the Hg, Cr and Cu were primarily derived from industrial activities, and the Pb originated from traffic pollution. The Hg in the Baihua Reservoir might also have been associated with coal combustion pollution. An analysis of ecological risk using sediment quality guidelines showed that there were moderate toxicological risks for sediment-dwelling organisms in both reservoirs, mainly from Hg and Cr. Ecological risk analysis using the Hakanson index suggested that there was a potential moderate to very high ecological risk to humans from fish in both reservoirs, mainly because of elevated levels of Hg and Cd. The upstream Hongfeng Reservoir acts as a buffer, but remains an important source of Cd, Cu and Pb and a moderately important source of Cr, for the downstream Baihua Reservoir. This study provides a replicable method for assessing aquatic ecosystem health in adjacent plateau reservoirs.

## Introduction

There is worldwide concern about heavy metal contamination because of the environmental persistence of these elements, biogeochemical recycling and the ecological risks that metals present [1], [2]. Large numbers of anthropogenically generated heavy metals from urban areas, agricultural areas and industrial sites are discharged into aquatic environments where they are transported in the water column, accumulated in sediment, and biomagnified through the food chain [3], resulting in significant ecological risk to benthic organisms, fish and humans [4]. Sediments are the main sink for heavy metals in aquatic environments [5], and sediment quality has been recognized as an important indicator of water pollution [6]. However, heavy metals are not permanently bound to sediments [7], and they may be released into the water column when the environmental conditions change (e.g., temperature and pH) or when sediments undergo other physical or biological disturbances [8]. Furthermore, reservoir construction generally leads to an increase in residence time, resulting in high accumulations of heavy metals in sediments. Consequently, it is important to analyze sediments from reservoirs for heavy metals to support environmental management, particularly for sediments from drinking water reservoirs.

Understanding the sources of pollutants in aquatic sediments is important for pollution control. Statistical approaches, such as Pearson correlation analysis, principal components analysis (PCA), and cluster analysis, are considered to be effective tools for uncovering pollution sources and have been used successfully in many studies of heavy metal pollution in sediments [1], [2], [3], [7], [9], [10], [11]. Risk assessments of the environmental pollution are also critical for sediment analysis. The ecological risk of heavy metals in sediments differs for different receptors (e.g., sediment-dwelling organisms, fish or humans). The thresholds in sediment quality guidelines (SQGs) have been used to evaluate the potential adverse effects of heavy metals on sediment-dwelling organisms in freshwater systems [12], [13], [14], [15]. However, few SQGs have been developed to assess the adverse effects of heavy metals in sediment on higher trophic levels (fish or other wildlife) [16], [17]. The potential ecological risk index proposed by Hakanson [18] is based on heavy metal concentrations in sediment, and it is the simplest and most popular method for assessing the human health risk from fish consumption.

The rapid growth of urbanization and industrial development has resulted in increasing heavy metal pollution in the aquatic sediment of the Yunnan-Guizhou Plateau [11], [19]. Several cascade hydropower stations have been built along the region’s large rivers (e.g., Wujiang, Jinshajiang and Nanpanjiang) since the 1950s, and stations are still being built for electricity production today, leading to a continuous series of reservoirs along the rivers [20]. Previous studies have evaluated the carbon (C) cycle [21], [22] and the mercury (Hg) balance [20], [23] in adjacent plateau reservoirs, and have demonstrated how upstream reservoirs influence downstream reservoirs. However, little research has been conducted on other heavy metals in the adjacent reservoirs on this plateau. In addition, several decades after their construction, the functions of these reservoirs were changed, so they now supply drinking water to the human population, which has grown rapidly due to economic growth. Currently, these reservoirs are the main drinking water sources on the Yunnan-Guizhou Plateau. Although some pollution sources were closed or moved when the reservoir functions were changed, the residue of previous pollutants still remains in reservoir sediments. Furthermore, the Yunnan-Guizhou Plateau is famous for its karst landforms, and the hydrogen carbonate (HCO_3_^−^) concentration and pH are both high in the aquatic environment [24], [25], [26]. The alkaline environment favors heavy metal accumulation in sediment [27], while the karst landform promotes interactions between groundwater and surface water through fractures (sinkholes, conduits and caves) or carbonate bedrock [26], [28], [29]. These interactions can complicate heavy metal transport and increase the ecological risk of secondary pollution. Therefore, it is important and necessary to investigate the heavy metal pollution and to assess the associated pollution sources and ecological risks from reservoir sediments on the Yunnan-Guizhou Plateau, particularly for drinking water reservoirs. The objectives of this paper are to (1) identify the pollution sources of heavy metals in the sediment from two adjacent drinking water reservoirs on the Yunnan-Guizhou Plateau, (2) estimate the associated ecological risk by considering different receptors, and (3) discuss the influence of upstream reservoirs (as buffers or sources of heavy metals) on downstream reservoirs.

## Materials and Methods

### Study Areas

**Figure 1:**
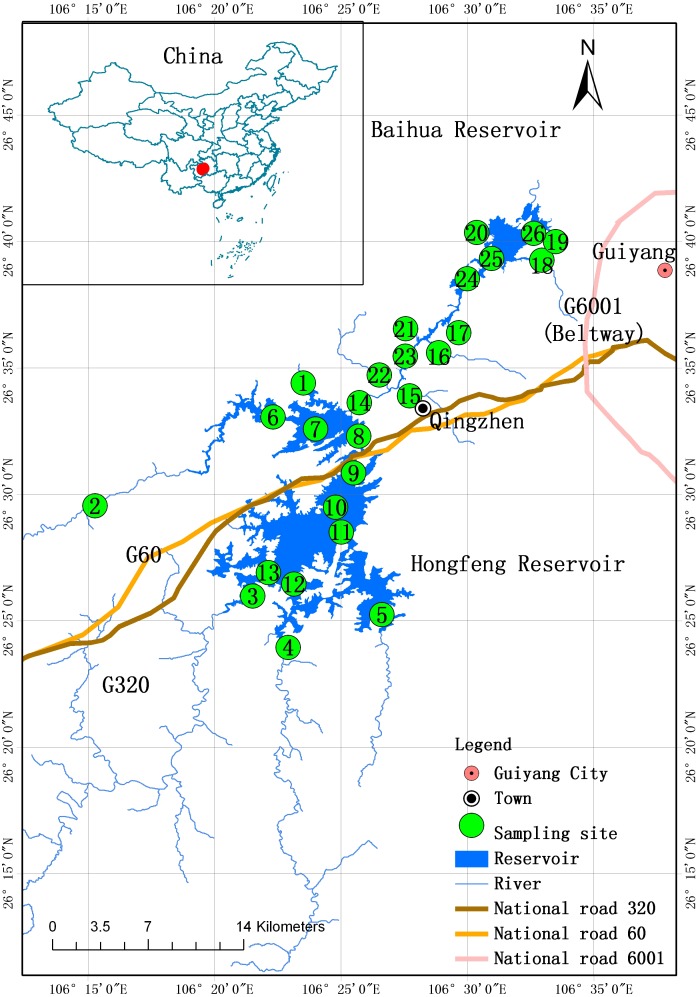
Map of Hongfeng and Baihua Reservoirs on the Yunnan-Guizhou Plateau, Southwest China.

The Hongfeng and Baihua Reservoirs are two adjacent reservoirs on the Yunnan-Guizhou plateau, just northwest of Guiyang City, the capital of Guizhou Province, Southwest China (Fig. 1). These two reservoirs were constructed on the main channel of the Maotiao River, a branch of the Wujiang River in the Yangtze River Basin, in 1958 and 1960, respectively. The Maotiao River was one of the first rivers to be used for cascade hydropower in China. The Hongfeng is the first reservoir and the Baihua is the second of seven cascade hydropower stations along the Maotiao River. The Hongfeng Reservoir covers a water surface area of 57.2 km^2^, while the Baihua Reservoir covers an area of 14.5 km^2^. Both reservoirs are very deep, with each having a maximum depth of approximately 45 m. The Hongfeng Reservoir consists of the North and South Lakes (which have different flow directions, Fig. 1), and has five main inflows, two into the North Lake and three into the South Lake. The Maotiao River is the only outlet of the Hongfeng Reservoir, and also serves as the major inlet of the Baihua Reservoir. The Baihua Reservoir has eight additional minor inflows and one outlet. For their first 30 years, the reservoirs were mainly used for electricity generation and flood control. During this period, as industry, agriculture, tourism and fishery production were established and developed in the basin, the water quality in both reservoirs declined. However, because of an increasing demand for water through the 1990s, the two reservoirs were designated as drinking water sources for Guiyang City. The major function of both reservoirs was changed to drinking water supply in 2000, at which point the government strengthened their environmental protection. The pollution sources have gradually decreased, but the sediments may still hold residue from earlier pollution.

**Figure 2:**
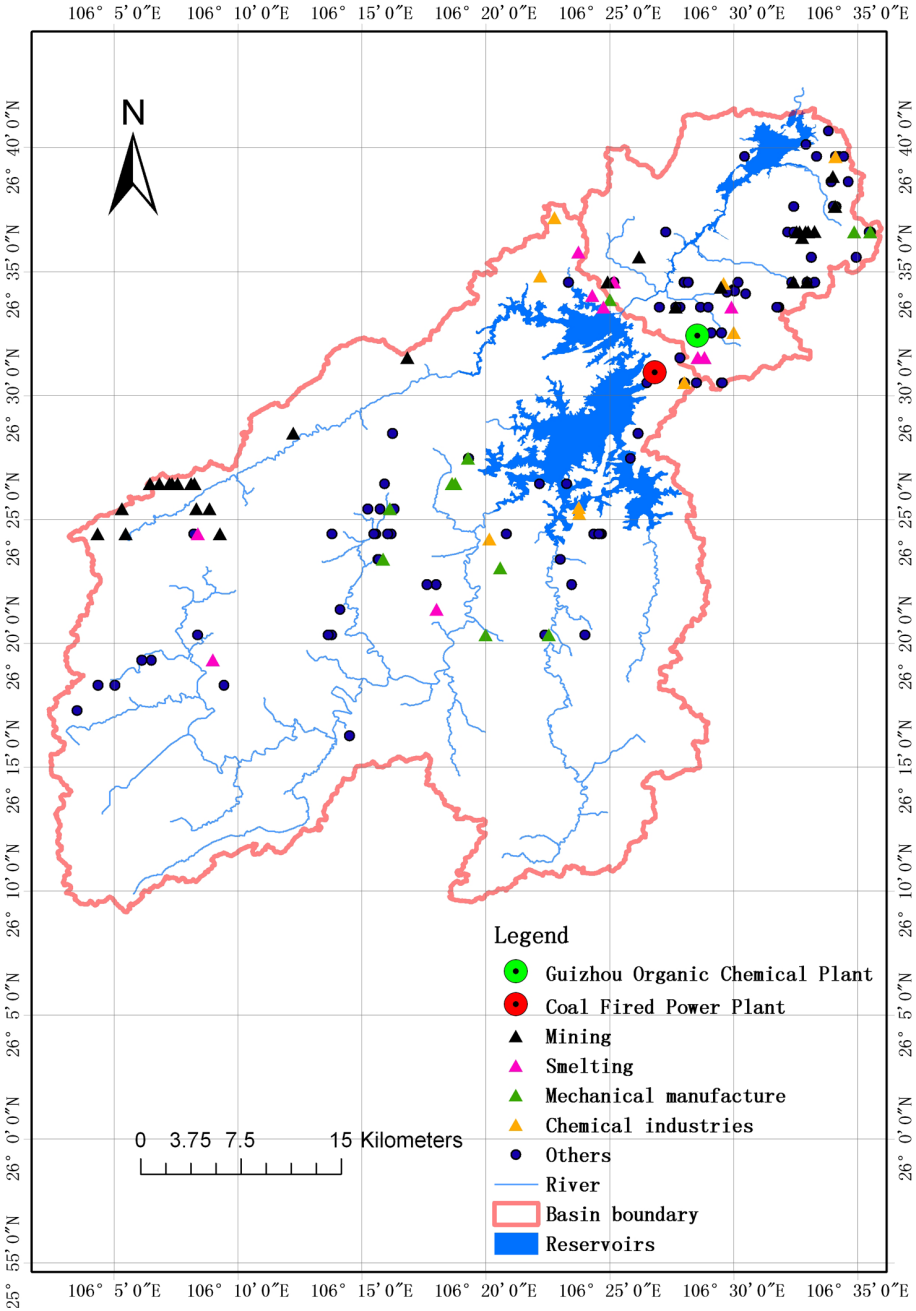
Distribution of main point sources of pollution in the Hongfeng and Baihua Reservoir basins.

Heavy metal pollution is one of the most prominent environmental problems in the study reservoirs, mainly owing to intense anthropogenic activities. Figure 2 shows the distribution of main point sources in Hongfeng and Baihua Reservoirs basin from the first China pollution source census in 2008 (provided by the Guiyang Research Academy of Environmental Sciences). There are many mining, smelting, mechanical manufacture, chemical and other industries (e.g., building material, food and pharmaceutical factories) in the catchment area of the Hongfeng Reservoir (1596 km^2^), which are major sources of heavy metals (Fig. 2). There is also a large coal-fired power plant (300 MW) situated on the southeast bank of the Hongfeng Reservoir [30], which is the main source of atmospheric deposition, especially for Hg and other heavy metals associated with coal combustion. The catchment area of the Baihua Reservoir is 1895 km^2^, but pollutants are first transported into the Hongfeng Reservoir, which may serve as a buffer for the Baihua Reservoir. The Baihua Reservoir receives direct inputs from an area of only 299 km^2^. Even so, there are many intense point sources in this small area, including various heavy and light industries (Fig. 2), which have resulted in serious heavy metal pollution in the Baihua Reservoir. In particular, the Baihua Reservoir is noted for its Hg contamination from the Guizhou Organic Chemical Plant (GOCP) [31], which is located in the upper reaches of the Baihua Reservoir and downstream of the Hongfeng Reservoir. The GOCP used Hg-based technology to produce acetaldehyde and discharged Hg-laden wastewater to the Baihua Reservoir via the Dongmenqiao River until 1997. The pollution caused by the GOCP persists to the present day.

### Sampling and Analysis

Two field surveys were conducted in December 2010 and April 2012. Water and sediment samples were collected from 26 sites in the Hongfeng and Baihua Reservoirs (Fig. 1 and Table 1). The field studies were permitted by the Administration of Hongfeng, Baihua and Aha Reservoirs, and did not involve endangered or protected species. There were 13 sites in each reservoir, comprising inlets of main tributaries (sites 1–5 in the Hongfeng Reservoir and sites 14–22 in the Baihua Reservoir) and representative sites within both reservoirs (sites 6–13 in the Hongfeng Reservoir and sites 23–26 in the Baihua Reservoir). Surface water samples were collected in acid-washed polyethylene sample bottles and were acidified with 1∶1 nitric acid: deionized water. Water samples were stored at 4°C immediately upon returning from the field. The upper 0–10 cm of sediment was collected, placed into pre-cleaned polyethylene bags, and taken to the laboratory. All sediment samples were freeze-dried and passed through a 2 mm nylon sieve to discard the coarse debris. A pestle and mortar was then used to grind the sieved sediments until all particles were fine enough to pass through a 0.147 mm nylon sieve. Sediment samples were digested in a microwave digestion system with a HNO_3_-HF-HClO_4_-HCl acid mixture solution before analysis for total heavy metal content. All water samples and the solutions of the digested sediment samples were analyzed by inductively coupled plasma atomic emission spectroscopy for Cr, Cu and Pb. Cd concentrations were determined by graphite furnace atomic absorption spectrophotometry. As and Hg were measured using atomic fluorescence spectrometry. Quality assurance and quality control of the analyses processes were assessed by duplicates, method blanks and standard reference materials [11].

**Table 1:** Locations of sampling sites in the Hongfeng and Baihua Reservoirs on the Yunnan-Guizhou Plateau, Southwest China.

Reservoir	Site	Longitude	Latitude	Description	
Hongfeng Reservoir	1	106°23′30.64″	26°34′24.52″	At main tributaries	Maibao River
	2	106°15′3.58″	26°29′22.80″		Maiweng River
	3	106°21′30.89″	26°25′58.34″		Yangchang River
	4	106°22′54.88″	26°23′56.79″		Maxian River
	5	106°26′42.01″	26°25′4.80″		Houliu River
	6	106°22′11.10″	26°33′44.07″	Within reservoir	Taipingdi
	7	106°24′2.05″	26°32′34.80″		Center of the North Lake
	8	106°23′17.43″	26°32′27.13″		Junction of the Northand South Lakes
	9	106°26′07.06″	26°30′58.03″		Houwu
	10	106°24′39.31″	26°29′19.07		Center of the South Lake
	11	106°25′04.17″	26°28′33.89″		Jiangjundong
	12	106°23′01.81″	26°26′26.73″		Yangjiajun
	13	106°22′01.22″	26°26′37.57″		Sanjiazhai
Baihua Reservoir	14	106°25′43.88″	26°33′37.65″	At main tributaries	Outlet of Hongfeng Reservoir
	15	106°27′19.60″	26°34′7.06″		Dongmenqiao River
	16	106°28′51.88″	26°35′36.16″		Maicheng River
	17	106°29′39.21″	26°36′23.43″		Dianzishanggou River
	18	106°32′56.81″	26°39′15.61″		Maixi River
	19	106°33′16.38″	26°40′6.00″		Banpochanggou River
	20	106°30′22.33″	26°40′21.27″		Maolizhaigou River
	21	106°27′33.58″	26°36′32.72″		Xiaohekou River
	22	106°26′47.46″	26°34′17.11″		Changchong River
	23	106°27′25.20″	26°35′27.60″	Within reservoir	Huaqiao
	24	106°30′4.42″	26°38′31.42″		Xuantiandong
	25	106°30′50.23″	26°39′1.37″		Tangerpo
	26	106°32′48.39″	26°40′18.16″		Chafan

### Spatial and Statistical Analyses

Spatial and statistical analyses were performed by using Arc GIS 9.3 and SPSS 17.0 for Windows (SPSS Inc., Chicago, IL) to investigate the heavy metal pollution sources separately for the Hongfeng and Baihua Reservoirs. A one-way ANOVA was performed on heavy metal concentrations in sediment to determine whether the differences between the two field surveys were significant. Pearson correlation analysis was used to determine the relationships between the heavy metals in sediment. To obtain more reliable information about the relationships between the heavy metals, a PCA with Varimax normalized rotation was performed separately for the Hongfeng and Baihua Reservoirs. The PCA calculated eigenvectors to determine the common pollution sources, and components with eigenvalues greater than 1 were considered to be relevant [32]. Components with factor loadings above 0.75, between 0.5 and 0.75, and between 0.3 and 0.5 were considered to be strong, moderate and weak, respectively [33]. Boxplot is a convenient way to depict the full range of data and compare the distributions among different datasets. In this study, the boxplot was used to compare the heavy metal concentrations at site 14, the outlet of the Hongfeng Reservoir and inlet of the Baihua Reservoir, with concentrations at other sites in the tributaries and at sites within the reservoirs, in order to discuss the influence of the upstream Hongfeng Reservoir on the downstream Baihua Reservoir.

### Ecological Risk Assessment

We used two methods to assess the ecological risk of the heavy metals in surface sediments to benthic organisms and humans. First, we used the consensus-based SQGs for freshwater ecosystems that were proposed by MacDonald et al. [15], which included a threshold effect concentration (TEC) and a probable effect concentration (PEC). TECs are the concentrations below which adverse effects are not expected on sediment-dwelling organisms, while PECs are concentrations above which adverse effects are expected to occur frequently [34], [35]. The mean PEC quotient (*m-PEC-Q*) [36] was also calculated for each sediment sample to assess the biological significance of the contaminant mixtures as follows:
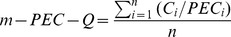
(1)where *C_i_* is the sediment concentration of compound *i*, *PEC_i_* is the PEC for compound *i* and *n* is the number of compounds *i*. Four ranges of the mean PEC quotient were developed by Long et al. [36] for ranking samples in terms of toxicity incidence (Table 2).

**Table 2:** Ecological risk assessment criteria for the sediment quality guidelines (SQGs) and Hakanson index.

Method	*C* or *E_r_^i^*	Potential ecological risk for single heavy metal	m-PEC-Q or *RI*	Ecological risk for all Heavy metals
SQGs	*C*<TEC	Low	m-PEC-Q<0.1	Low (<14%)^a^
	TEC <*C*<PEC	Moderate	0.1< m-PEC-Q <1.0	Moderate (15–29%)^a^
	*C*>PEC	High	1.0<m-PEC-Q<5.0	Considerable (33–58%)^a^
			m-PEC-Q >5.0	Very high (75–81%)^a^
Hakanson index	*E_r_^i^<40*	Low	*RI<95*	Low
	40<*E_r_^i^*<80	Moderate	*95<RI<190*	Moderate
	80<*E_r_^i^*<160	Considerable	*190<RI<380*	Considerable
	160<*E_r_^i^*<320	High	*RI>380*	Very high
	*E_r_^i^>*320	Very high		

*C:* concentration of heavy metal in surface sediment.

TEC: threshold effect level; PEC: probable effect level [15].

m-PEC-Q: mean PEC quotient; ^a^incidence of toxicity [36].

We also used the Hakanson index, which reflects the risk to human health from fish consumption. This index is based on the assumption that the sensitivity of the aquatic system depends on its productivity [3], [18]. The potential ecological risk index (*RI*) was introduced to evaluate heavy metal pollution in sediments by considering the toxicity of heavy metals and the environmental response. The *RI* is calculated as follows:
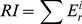
(2)

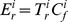
(3)

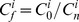
(4)where *RI* is the total potential ecological risk index for multiple metals, *E_r_^i^* is the potential ecological risk index for a single metal, and *T_r_^i^* is the toxic-response factor for a given metal, considering both toxicity and the sensitivity. *C_f_^i^* is the contamination factor, *C_0_^i^* is the metal concentration in the sediment and *C_n_^i^* is a reference value for metals. In this study, because both reservoirs are moderately eutrophic [37], *T_r_^i^* was described as Hg (40) > Cd (30) > As (10) > Cu = Pb (5) > Cr (2), based on the assumption that the bioproduction index was 5 [18]. *C_n_^i^* was defined as the upper limit of the natural background value for a given metal in the study area (Table 3). Four ranges of the risk factor *RI* were suggested by Hakanson, based on eight metals (polychlorinated biphenyls (PCBs), Hg, Cd, As, Pb, Cu, Cr, and zinc (Zn)). PCBs and Zn were not considered in this study. Based on the different contributions of these elements to the ecological risk index *RI*, the adjusted evaluation criteria for *RI* based on the six metals in this study are listed in Table 2.

**Table 3:** Summary statistics for heavy metal concentrations in surface sediments from the Hongfeng and Baihua Reservoirs.

		Hg	Cd	Pb	Cr	Cu	As	
HongfengReservoir	Mean (Min, Max)	0.32(0.08, 1.03)	0.28(0.01, 0.85)	28.41(0.10, 89.20)	86.91(34.10, 141.00)	43.50(15.70, 93.60)	15.33(0.12, 45.54)	This study (2010.12)
	S.D.	0.26	0.36	27.55	29.65	25.09	14.92	
	CV(%)	82.86	130.78	96.97	34.12	57.67	97.31	
	Mean (Min, Max)	0.27(0.04, 0.56)	0.53(0.31, 1.37)	30. 22(1.21, 89.20)	82.78(41.00, 141.00)	45.46(23.20, 73.80)	23.31(17.31, 29.61)	This study (2012.4)
	S.D.	0.17	0.27	25.63	23.45	17.72	3.98	
	CV(%)	62.33	51.27	84.78	28.32	38.99	17.07	
	2007.3	0.99		34.31		89.11	49.90	Huang et al. [40]
	2008.8			58.39	120.16	69.84		Zeng et al. [41]
	2008.10	0.66	0.77	35.91	87.98	91.85	29.74	Liu et al. [42]
	2009.5	0.46	0.65		118.00	88.00	40.80	He et al. [27]
Baihua Reservoir	Mean (Min, Max)	0.68(-^a^, 2.20)	0.58(0.01, 1.00)	27.28(0.10, 51.90)	76.24(30.80, 143.00)	36.71(0.36, 65.90)	29.95(7.95, 48.19)	This study (2010.12)
	S.D.	0.73	0.37	16.89	31.50	19.77	14.80	
	CV(%)	108.24	64.15	61.90	41.31	53.86	49.42	
	Mean (Min, Max)	0.45(0.01, 1.25)	0.61(0.23, 1.00)	27.84(0.10, 51.90)	76.38(30.12, 143.10)	43.16(9.38, 73.55)	26.23(7.95, 34.75)	This study (2012.4)
	S.D.	0.48	0.28	18.15	31.45	21.49	7.30	
	CV(%)	107.21	46.13	65.19	41.17	49.79	27.82	
	2007	18.90	0.88	16.05	59.75	74.97	53.34	Huang [39]
	2010.5		0.95	38.90	66.00	67.50		Tian et al. [43]
Natural Background Value		0.08–0.15	0.08–0.12	18.50–23.90	73.90–94.60	27.30–36.70	27.00–50.00	NEPA [44]

All concentrations are in mg/kg dry weight. ^a^-: not detected. S.D.: standard deviation; CV: coefficients of variation.

## Results and Discussion

### Heavy Metal Concentrations in Water Samples and Surface Sediments

Of the 6 heavy metals, only Hg and As were detected in the water samples in December 2010, while Hg, Cd, Cr (VI) and As were detected in April 2012. Their concentrations were similar in the Hongfeng and Baihua Reservoirs, with Cd, Cr (VI), and As concentrations lower than Class I as defined in the Chinese Environmental Quality Standards for Surface Water (GB3838-2002, <0.001 mg/L for Cd, <0.01 mg/L for Cr (VI), and <0.05 mg/L for As) and Hg concentrations ranging from Class I (GB3838-2002, <0.00005 mg/L) to Class IV (GB3838-2002, 0.0001–0.001 mg/L) among different sites. The low heavy metal concentrations in water were primarily due to the accumulation in sediments because the alkaline environment in both reservoirs provides ideal conditions for adsorption and precipitation [27]. Moreover, the sediment accumulation rate in both reservoirs was quite high [38], contributing to the removal of heavy metals from the water column. A prior one-way ANOVA analysis was conducted to examine the variation in heavy metal concentrations in sediment between the two field surveys. None of the heavy metals in Hongfeng and Baihua Reservoirs displayed significant variation in means (*p*>0.05), although Cd in Hongfeng Reservoir and As in both reservoirs showed significant changes in their variances (*p*<0.05). The significant differences in variance for Cd and As are mainly because of their higher concentrations in the sites within the reservoirs and the reduced spatial heterogeneity in the second field sampling comparing to the first one. However, the general spatial patterns for Cd and As (with higher concentrations at sites in the tributaries than at sites within the reservoirs) were still similar between the two field surveys. Those results indicate that the pollution sources for the metals were relatively stable between the two surveys. The concentrations of heavy metals in surface sediments of both reservoirs from the two field surveys are summarized in Table 3. Heavy metal concentrations in sediment were much higher than those in water. In general, the mean Hg, Cd and As sediment concentrations in the Baihua Reservoir were higher than those in the Hongfeng Reservoir, while Pb, Cr and Cu were higher in the Hongfeng Reservoir. Comparison with the results of previous studies [27], [39], [40], [41], [42], [43] shows that most of the heavy metal concentrations in both reservoirs have decreased, though by differing amounts (Table 3). In particular, the Hg concentrations in the Baihua Reservoir have decreased significantly, indicating that measures taken in recent years have been effective and resulted in improvements. During the two field surveys, the mean concentrations of Hg, Cd, Pb and Cu, and the maximum concentrations of Cr in both reservoirs exceeded the upper limit of the natural background values for the study area [44], indicating anthropogenic sources. However, the concentrations of As (including the minimum and maximum values) in both reservoirs were well within the range of the natural background values [44], implying no significant anthropogenic impact and primarily lithogenic sources.

### Heavy Metal Pollution Sources

**Figure 3:**
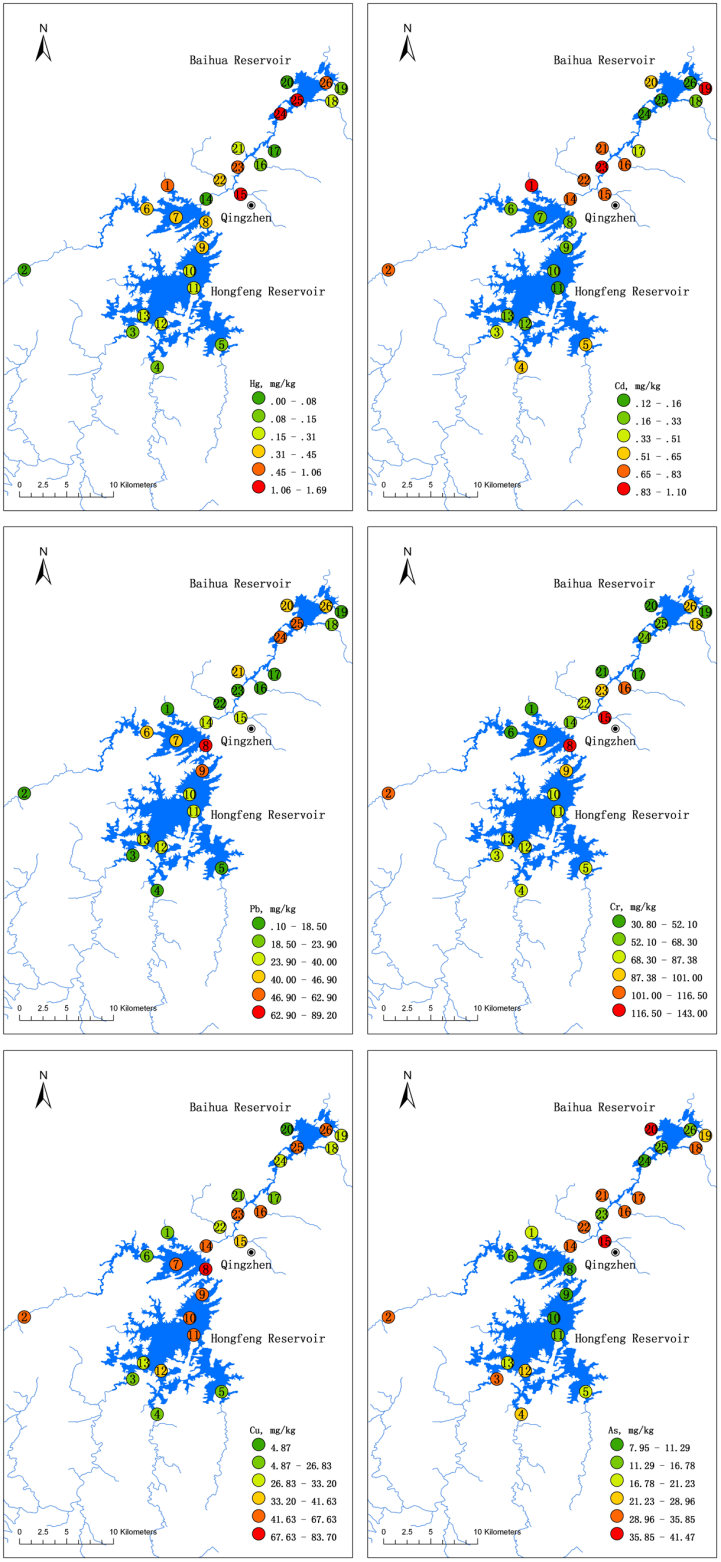
Heavy metal concentrations in surface sediments from Hongfeng and Baihua Reservoirs.

To develop control strategies for environmental pollution, it is very important to identify its source. Spatial and statistical analyses were performed to identify the possible pollution sources for heavy metals in the Hongfeng and Baihua Reservoirs. The average concentrations of heavy metals in the sediments from the two field surveys were used to study the spatial distributions, while all data in the sediments from the two field surveys were used in a Pearson correlation analysis and PCA. The spatial distribution patterns of Hg, Cd, Pb, Cr, Cu and As in surface sediments of both reservoirs are shown in Figure 3. The Pearson correlation coefficients and the results of the PCA for the investigated metals are shown in Table 4 and Table 5, respectively. All of the results were generally consistent with each other.

**Table 4:** Correlations between heavy metals in surface sediments from the Hongfeng and Baihua Reservoirs.

	Hg	Cd	Pb	Cr	Cu	As
Hg	**1.000**	**0.090**	**0.321**	−**0.234**	−**0.038**	−**0.343**
Cd	−0.422*	**1.000**	−0.540**	−**0.168**	−**0.237**	0.625**
Pb	0.511**	−0.506**	**1.000**	0.438*	0.595**	−0.524**
Cr	0.495*	0.013	−0.086	**1.000**	0.785**	**0.046**
Cu	0.241	−0.013	0.006	0.509**	**1.000**	−**0.183**
As	−0.414*	0.476*	−0.314	0.067	−0.333	**1.000**

Hongfeng Reservoir in the upper right corner (blod); Baihua Reservoir in the lower left corner.

Levels of significance: *p<0.05; **p<0.01.

**Table 5:** Principal Component Analysis (PCA) for heavy metals in surface sediments from the Hongfeng and Baihua Reservoirs.

Heavy metal	Hongfeng Reservoir			Baihua Reservoir	
	F1	F2	F3	F1	F2
Hg			0.973	−0.669	0.523
Cd		0.924		0.821	
Pb	0.605	−0.575	0.378	−0.800	
Cr	0.929				0.902
Cu	0.930				0.799
As		0.851	−0.334	0.705	
Variance (%)	35.29	32.24	21.35	37.84	29.32
Cumulative (%)	35.29	67.53	88.88	37.84	67.16

Factor loadings smaller than 0.3 have been removed.

Extraction method: PCA, Rotation method: Varimax with Kaiser normalization.

Specifically, the PCA yielded three significant components for Hongfeng Reservoir and two significant components for Baihua Reservoir, accounting for 88.88% and 67.16% of the cumulative variance, respectively (Table 5). For the Hongfeng Reservoir, the first component (F1), explaining 35.29% of the total variance, had strong positive loadings of Cr and Cu, and moderate positive loading of Pb. Those three heavy metals exhibited similar spatial distributions in the Hongfeng Reservoir, with unexpectedly higher concentrations at reservoir sites than at tributary sites. In particular, site 8 (at the junction of the North and South Lakes) showed the highest concentrations for all of the three heavy metals, and site 9 (near Houwu) also showed relatively high concentrations (Fig. 3). In addition, those three heavy metals were highly correlated (Table 4, p<0.01 for Cr-Cu and Cu-Pb, p<0.05 for Cr-Pb), indicating their similar origins or comparable chemical properties [45]. This phenomenon might be caused by two possible reasons. Firstly, Bai et al. [46] found that traffic pollution was responsible for the high heavy metal concentrations (including comparable Cr, Cu and Pb concentrations with our study) along the roadside of National Road 320 in the Yunnan province (adjacent to Guizhou Province), and Zhu et al. [47] also found that road dust samples were severely polluted by Cr, Cu and Pb in another metal smelting/processing industrial city in Guizhou Province. In this study, the National Road 60 and the National Road 320 pass close to the junction of the South and North Lakes of the Hongfeng Reservoir (Fig. 1), which suggests that the traffic emissions, through atmospheric deposition and road runoff, could result in heavier pollution in sites near the roadway (site 8 and 9). Secondly, the high concentrations of Cr, Cu and Pb at reservoir sites are likely to be related to the residual effect from former industrial activities (e.g., mining, smelting, mechanical manufacture and chemical industry). The metals are more likely retained in the sediment of sites within the reservoirs rather than sites in the tributaries because heavy metal accumulation in lake sediments is generally higher than that in rivers [3]. Additionally, the complex hydrodynamic conditions at the junction of the South and North Lakes may affect the heavy metal distributions in sediment, which requires further research. Therefore, the first component (F1) might reflect mixed sources from traffic pollution and the residual effect of former industrial influence. The second component (F2), explaining 32.24% of the total variance, was dominated by Cd and As. Similar spatial patterns were observed for Cd and As, with higher concentrations at tributary sites than at sites within the reservoirs, indicating that they mainly come from the inflows (Fig. 3). As expected, significantly positive correlations were found between Cd and As (Table 4, p<0.01). However, Cd showed apparent anthropogenic origin, with most sites exceeding its natural background values, while As levels suggested natural origins, with all sites well within the natural background values (Table 3). Cd is closely related to industrial activities, such as smelting, electroplating and plastics production in the upstream areas. Hence, F2 may reflect the pollution through inflows from both industrial activities and natural weathering and erosion. The third component (F3) had strong positive loading on Hg and a weak positive loading on Pb, accounting for 21.35% of the total variance. The highest Hg concentration in Hongfeng Reservoir was found at site 1 in the tributary of Maibao River, which has several smelting and chemical industries in its upstream (Fig. 2). Meanwhile, for both Hg and Pb, the North Lake were more polluted than the South Lake, and three tributaries in the South Lake showed concentrations well within the natural background values. Feng et al. [30] found that runoff due to soil erosion was the main source of Hg in sediment in the South Lake of the Hongfeng Reservoir. Thus, F3 may reflect the pollution from inflows from industrial activities in the North Lake and lithogenic origin in the South Lake. In addition, He [48] found that atmospheric deposition from coal combustion was also an important source of Hg in the Hongfeng Reservoir, which was not clearly distinguished by the PCA.

For Baihua Reservoir, the first component (F1), explaining 37.84% of total variance, showed strong positive loadings on Cd and As. Similar spatial distributions (with higher concentrations at tributary sites than at sites within the reservoirs) (Fig. 3) and positive correlations were also found between Cd and As (Table 4, p<0.05). As discussed above, F1 in Baihua Reservoir might be similar to F2 in Hongfeng Reservoir, including the pollution through inflows from both industrial activities and natural origin. The second component (F2) had a moderate positive loading of Hg and strong positive loadings on Cr and Cu. The highest Hg and Cr concentration in the Baihua Reservoir was found at site 15 in the Dongmengqiao tributary, which received wastewater from the GOCP and many other industries (Fig. 2). The highest Cu concentration in the Baihua Reservoir was at site 16 in the tributary of the Maicheng River, which has several industries (especially mining) in its upstream (Fig. 2). Strong associations were found between Cr and Cu (p<0.01) and between Cr and Hg (p<0.05) (Table 4). F2 obviously represented industrial activity upstream. On the other hand, the PCA failed to identify Pb sources in Baihua Reservoir, but only showed its strong negative associations with F1, indicating that there may be significant sources other than F1 and F2 for Pb. The spatial pattern of Pb in the Baihua Reservoir also showed much higher concentrations at sites within the reservoir than at sites in the tributaries (Fig. 3). Although no main road crosses Baihua Reservoir directly, it is close to the urban district of Guiyang City, which has several roads and high traffic density (Fig. 1 only shows the main beltway and there are many other crisscrossed roads inside the beltway). In addition, Pb was added to gasoline in China until June 2000 [49]. Hence, Pb may originate from traffic pollutants deposited atmospherically. Moreover, highly positive correlations were found between Pb and Hg (p<0.01), implying that Hg might also come from atmospheric deposition (coal combustion) in addition to from the factors associated with F2.

### Ecological Risk Assessment of Heavy Metals in Surface Sediments

**Figure 4:**
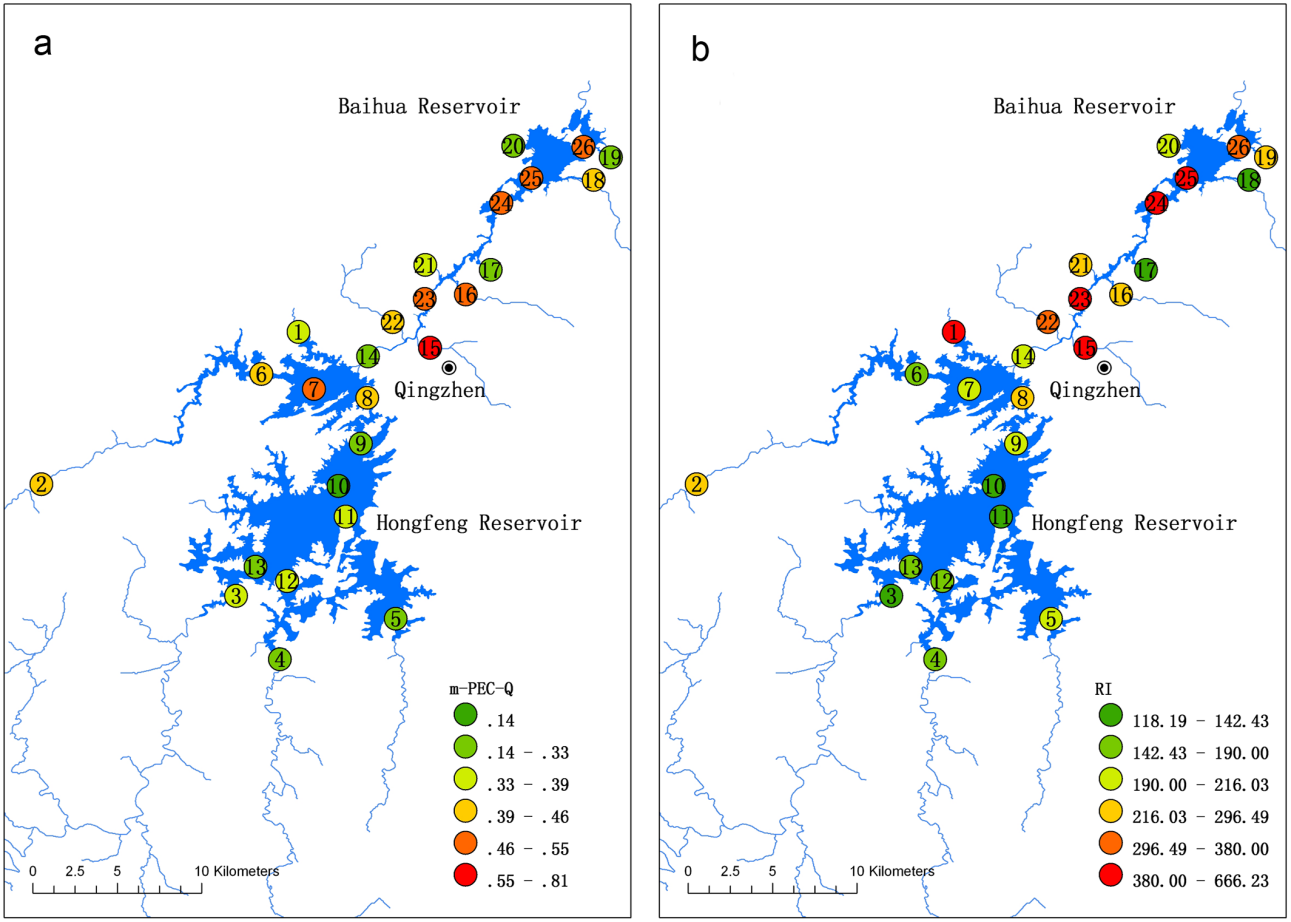
Mean PEC quotient (a) and potential ecological risk indexes (b) of heavy metals in sediments.

Because there was no significant change in the mean heavy metal concentrations in sediment between the two field surveys, the average concentrations were used to study the ecological risk of single heavy metals and the combined ecological effects of six heavy metals for the two study reservoirs using both the SQG and Hakanson index methods (Table 6). The SQG method revealed that the metal concentrations were within the TEC and PEC ranges for Hg, Cd, Pb and Cu at 69.23%, 7.69%, 38.46% and 61.54% of the sites in the Hongfeng Reservoir, and for Cd, Pb and Cu at 7.69%, 38.46% and 46.15% of sites in the Baihua Reservoir. The heavy metals at the remaining sites in the corresponding reservoirs fell below the TEC. Cr and As exceeded the PEC at 15.38% of sites in the Hongfeng Reservoir and Hg, Cr and As exceeded the PEC at 30.77%, 7.69% and 38.46%, respectively, of sites in the Baihua Reservoir. Previous studies have shown that ecological risk assessments should consider the regional background values and that exceeding the SQG values does not always lead to adverse ecological effects [50]. Therefore, the As concentrations within background ranges in both reservoirs should be excluded. The Cr in the Hongfeng Reservoir and both Hg and Cr in the Baihua Reservoir may pose significant ecological risks for sediment-dwelling organisms, and they deserve special attention. Heavy metals within the TEC–PEC (viz., Hg in Hongfeng Reservoir and Cd, Pb and Cu in both reservoirs) are also a cause for concern because this uncertain area may be considered to be moderately polluted [10]. The toxicity, derived from mean PEC quotients, that results from the mixture of the six heavy metals at each sampling site in both reservoirs is shown in Figure 4a. Overall, mean PEC quotients for samples in the Baihua Reservoir (range 0.28–0.81) were slightly higher than those in the Hongfeng Reservoir (range 0.14–0.55). However, the mean PEC quotients for all of the samples in both reservoirs were well within the range of 0.1 to 1.0, indicating moderate toxicological risks for sediment-dwelling organisms, with a toxicity incidence of between 15 and 29% in the study areas (Table 2).

**Table 6:** Results of ecological risk assessments for single heavy metal from two methods for the Hongfeng and Baihua Reservoirs.

		Hg	Cd	Pb	Cr	Cu	As
TEC		0.18	0.99	35.8	43.4	31.6	9.79
PEC		1.06	4.98	128	111	149	33
HongfengReservoir	% samples which exceeded TEC	69.23	7.69	38.46	92.31	61.54	84.62
	% samples which exceeded PEC	0.00	0.00	0.00	15.38	0.00	15.38
	% samples with *E_r_^i^*<40	30.77	7.69	100.00	100.00	100.00	100.00
	% samples with 40<*E_r_^i^*<80	23.08	53.85	0.00	0.00	0.00	0.00
	% samples with 80<*E_r_^i^*<160	38.46	23.08	0.00	0.00	0.00	0.00
	% samples with 160<*E_r_^i^*<320	7.69	15.38	0.00	0.00	0.00	0.00
	% samples with *E_r_^i^>*320	0.00	0.00	0.00	0.00	0.00	0.00
BaihuaReservoir	% samples which exceeded TEC	53.85	7.69	38.46	84.62	46.15	92.31
	% samples which exceeded PEC	30.77	0.00	0.00	7.69	0.00	38.46
	% samples with *E_r_^i^*<40	38.46	23.08	100.00	100.00	100.00	100.00
	% samples with 40<*E_r_^i^*<80	15.38	0.00	0.00	0.00	0.00	0.00
	% samples with 80<*E_r_^i^*<160	7.69	15.38	0.00	0.00	0.00	0.00
	% samples with 160<*E_r_^i^*<320	15.38	61.54	0.00	0.00	0.00	0.00
	% samples with *E_r_^i^>*320	23.08	0.00	0.00	0.00	0.00	0.00

All concentrations are in mg/kg dry weight.

The Hakanson method expresses the threat to humans from fish consumption. The results from this index were quite different from those for the SQG method (Table 6). Both Hg and Cd posed high potential ecological risks at 7.69% and 15.38% of sites, considerable risks at 38.46% and 23.08% of sites and moderate risks at 23.08% and 53.85% of sites, respectively, in the Hongfeng Reservoir. The risks were higher in the Baihua Reservoir, in which there was a very high potential ecological risk from Hg at 23.08% of sites, high risks from Hg and Cd at 15.38% and 61.54% of sites, considerable risks from Hg and Cd at 7.69% and 15.38% of sites, and a moderate risk from Hg at 15.38% of sites. However, the other heavy metals (Pb, Cr, Cu and As) posed little potential ecological risks for all sites in both reservoirs, with *E_r_^i^* values lower than 40. The high concentrations and toxic-response factors of Hg and Cd in both reservoirs contribute to their posing higher ecological risks than the other metals we examined. *RI* illustrates the potential ecological risk from heavy metal mixtures, and *RI* at all sites in both reservoirs were higher than 95 (Fig. 4b). Site 1 showed the highest potential ecological risk (*RI* = 484.25) in the Hongfeng Reservoir, at a level that should cause concern because it poses a very high risk. Sites 2, 5 and 7–9 exhibited considerable ecological risks, while other sites showed moderate ecological risks in the Hongfeng Reservoir. The combined ecological risk was more severe in the Baihua Reservoir. The *RI* at site 15 (Dongmenqiao tributary) and 23–25 (within the reservoir) were much higher than 380, which indicates a very high potential ecological risk. All of the other sites exhibited considerable ecological risks (except for the moderate ecological risks at site 17 and 18). Therefore, there are moderate to very high potential ecological risks from heavy metal mixtures in the sediments of both reservoirs. In addition, the contribution of the monomial potential ecological risk to *RI* for the six heavy metals in both reservoirs decreased in the following order: Hg ≈Cd > As > Cu >Pb > Cr, with the greatest ecological risk from Hg and Cd.

Overall, the ecological risks from either a single heavy metal or from mixed heavy metals were different for the two receptors (viz., sediment-dwelling organisms and human beings through fish consumption) in both prior contaminants and risk level. However, hot spots with higher ecological risks were similar even though two different methods were used, and they were mainly located in the North Lake and the Houwu area of the Hongfeng Reservoir and in the key tributaries and at all of the sites in the Baihua Reservoir. Therefore, the need for industrial wastewater and mining tailings treatment in upstream watersheds of both reservoirs should be highlighted, especially for the tributaries in the North Lake of the Hongfeng Reservoir and in the key tributaries of the Baihua Reservoir. Additionally, given that the lakes are sources for drinking water, continuous monitoring should be increasingly implemented in areas near their inflows. Finally, there is uncertainty in both the SQG and Hakanson index methods because the SQGs were developed in North America and the toxic-response factor in the Hakanson method is not very sophisticated. Therefore, further on-site or laboratory toxicological experiments should be carried out to ascertain the actual adverse effects on sediment-dwelling organisms and different fish species [10], [50], as well as to determine the impacts on human health from consuming fish from the study area.

### Influence of the Hongfeng Reservoir on the Baihua Reservoir

**Figure 5:**
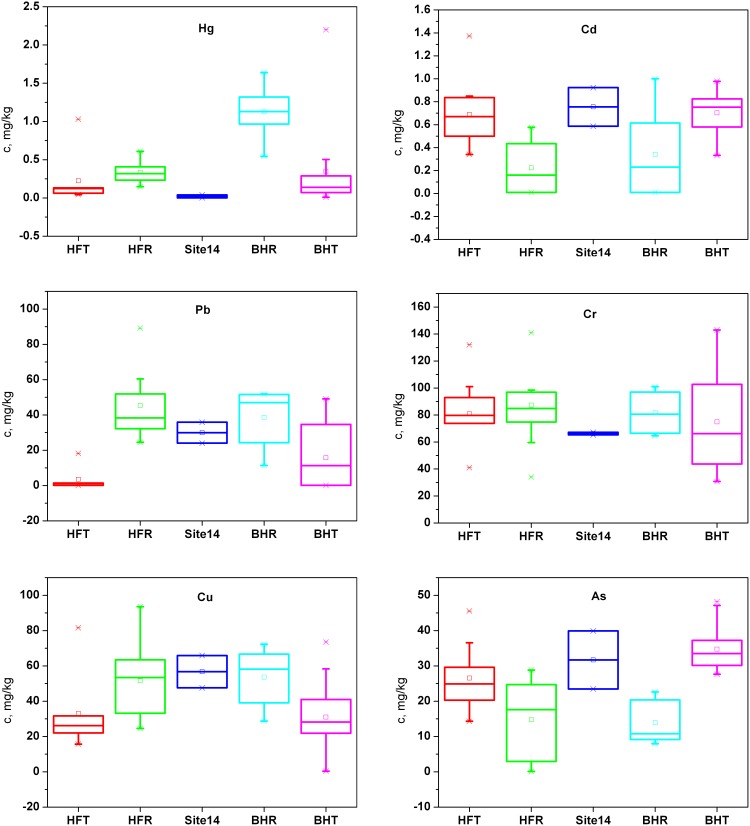
Comparison of heavy metal concentrations in sediments. (HFT: sites at inlets of main tributaries in the Hongfeng Reservoir, namely sites 1–5; HFR: representative sites within Hongfeng Reservoir, namely sites 6–13; BHT: sites at inlets of main tributaries in the Baihua Reservoir (except site 14), namely sites 15–22; BHR: representative sites within the Baihua Reservoir, namely sites 23–26).

Reservoir construction generally leads to an increase in residence time and a decrease in suspended solids and turbidity. For an alkaline reservoir on the Yunnan-Guizhou Plateau such as the Hongfeng Reservoir, heavy metals tend to be adsorbed to suspended solids, and sediments then settle on the lake bed, resulting in fewer heavy metals in water. Hence, in heavily polluted areas, reservoirs may serve as a sink for pollutants and a buffer for downstream receiving areas. In this study, the catchment area upstream of the Hongfeng Reservoir occupies 84% of the Baihua Reservoir catchment area, and the heavy metal concentrations at site 14, the outlet of the Hongfeng Reservoir and inlet of the Baihua Reservoir, reflect the buffering effect of the Hongfeng Reservoir according to our two field surveys. The metals (Hg, Cd, Cr (VI) and As) in the surface water at site 14 had lower concentrations than they did at the inflow tributary sites of the Hongfeng Reservoir (results not shown). The sediment concentration of Hg was much lower at site 14 than at most sites at the tributaries and within the Hongfeng Reservoir (Fig. 5). He [48] also found that the Hongfeng Reservoir functioned as a net sink for Hg and that it intercepted a large amount of Hg before it was conveyed to the Baihua Reservoir. The sediment concentrations of Cd and As were lower at site 14 than the maximum concentration at the tributaries of Hongfeng Reservoir, although they were generally higher there than at sites within Hongfeng Reservoir (Fig. 5). Due to the different pollution sources (some indirect rather than through inflows), higher concentrations of Pb, Cr and Cu were found at the reservoir sites than at the tributary sites, and the sediment concentrations of Pb, Cr and Cu were generally lower at site 14 than at sites within the Hongfeng Reservoir (Fig. 5). On the other hand, the outflow of the Hongfeng Reservoir has accounted for an average of 70% of the total inflow of the Baihua Reservoir over the last 6 years (2005–2010, provided by the Administration of Hongfeng, Baihua and Aha Reservoirs), implying that the Hongfeng Reservoir may also serve as an important source for total metals in the Baihua Reservoir. For example, the concentrations of the metals (Hg, Cd, Cr (VI) and As) detected in water samples at site 14 fell in the mid-range of the concentrations in the other tributaries of the Baihua Reservoir (results not shown). The concentrations of all of the heavy metals in sediments at site 14 were generally within the concentration ranges of heavy metals in other tributaries of the Baihua Reservoir, with Hg at the low end, Cr in the medium range, and Cd, Pb, Cu and As at the high end (Fig. 5). However, the sediment concentrations of Cd and As at site 14 were generally higher than at sites within the Hongfeng Reservoir. This pattern was not found in the other heavy metals, resulting in uncertainty when considering whether the relatively high concentrations of Cd and As at site 14 came from the Hongfeng Reservoir or not (Fig. 5). Because Cd was mainly from industrial activities and no emission sources exist near site 14 (as determined by the field investigation), the high concentrations at site 14 might result from the pollution of site 1 (Fig. 3) because our sampling sites within the Hongfeng Reservoir did not cover the area near the outlet. In terms of the spatial distribution and the lithogenic source of As, the high concentrations at site 14 might be affected by other factors, such as soil type and land use of the nearby banks. Therefore, for heavy metal concentrations in sediment, the Hongfeng Reservoir might be an important source of Cd, Cu and Pb, and a moderately important source of Cr, but might not be an important source of Hg and As for the Baihua Reservoir. The results also indicate that the Hongfeng Reservoir is not always the most important source for total metals in the Baihua Reservoir, and other tributaries contribute large quantities of pollutants to the Baihua Reservoir, some of which even exceed the levels in the Hongfeng Reservoir. It should also be noted that our field surveys only indirectly reflect the potential long-term impacts from Hongfeng Reservoir on heavy metals in the sediment of the Baihua Reservoir, and continuous monitoring of inflows and outflows of both reservoirs are needed for the specific contribution of the upstream reservoir to the downstream reservoir in future studies.

Other factors contribute to the adverse effects of the Hongfeng Reservoir on the Baihua Reservoir. The Hongfeng Reservoir is a deep reservoir with thermal stratification from May to November [37], and its release water is mainly from the hypolimnion, which has a lower DO concentration, higher CO_2_ concentration and lower pH than surface water during those months [21], [37]. The water chemistry in the hypolimnion favors the release of heavy metals from the sediments and changes the speciation and toxicity of heavy metals. He et al. [37] found that the low DO and pH in hypolimnion accelerated Hg methylation at Houwu (near site 9) and enhanced the release of methylmercury from sediments at Daba (near the outlet) in the Hongfeng Reservoir in summer. He et al. [37] also concluded that the Hongfeng Reservoir was a net source of methylmercury for the Baihua Reservoir. In addition, the release of hypolimnetic water has a cooling effect in the summer and a warming effect in winter, which may have a significant influence on the temperature downstream, and thus may indirectly influence the heavy metal distribution. Therefore, the outflow of the Hongfeng Reservoir may pose serious risks to ecosystems in the Baihua Reservoir. Further research is needed to help understand the influence of heavy metals and their chemical forms as they are transported downstream from reservoirs.

## Conclusions

This study of heavy metal (Hg, Cd, Pb, Cr, Cu and As) concentrations in surface water and sediments from two adjacent drinking water reservoirs (the Hongfeng and Baihua Reservoirs) on the Yunnan-Guizhou Plateau, Southwest China, showed that surface water was polluted by Hg, and sediments were polluted by Hg, Cd, Pb, Cr and Cu. In both reservoirs, Cd and As mainly came from industrial activities and lithogenic source through inflows, respectively. The Pb, Cr and Cu in Hongfeng Reservoir may have arisen from a mixture of sources (traffic pollution and residual effect of former industrial influence), and they were present at higher concentrations at the junction of the North and South Lakes. Hg sources in the Hongfeng Reservoir might include the sources that contribute Hg through inflows, which were different for the North (industrial activities) and South Lakes (lithogenic origin), and atmospheric deposition resulting from coal combustion. For the Baihua Reservoir, Hg, Cr and Cu were primarily derived from upstream industrial activities, and the Pb originated from traffic pollution. Additionally, the Hg in Baihua Reservoir might have come from atmospheric deposition (coal combustion). Ecological risk was assessed using the SQGs and the Hakanson potential ecological risk index. There were moderate toxicological risks for sediment-dwelling organisms (with the main risks from Hg and Cr) and moderate to very high potential ecological risks for humans from fish consumption (with the main risk coming from Hg and Cd) in both reservoirs. Overall, the risks were higher in the Baihua Reservoir. Improved treatment of industrial wastewater and mining tailings in upstream watersheds would alleviate the pollution and ecological risk in both reservoirs, especially for tributaries of the North Lake of the Hongfeng Reservoir and the key tributaries of the Baihua Reservoir. Ecological restoration could be considered to counteract the residual effects from previous pollution; however, more research is needed in this area. In terms of heavy metal concentrations, the Hongfeng Reservoir acts as a buffer, but it is still an important source of Cd, Cu and Pb and a moderately important source of Cr for the Baihua Reservoir. The Hongfeng Reservoir also had adverse effects on the Baihua Reservoir and merits further research. These findings provide useful information about sediment quality in adjacent reservoirs on the Yunnan-Guizhou Plateau.
